# Surgery for stage IIB–IIIB small cell lung cancer

**DOI:** 10.1186/s12957-023-03196-2

**Published:** 2023-10-23

**Authors:** Zhida Huang, Yue Liu, Suyu Wang, Kaixing Ai, Peng Zhang

**Affiliations:** 1 The Second Affiliated Hospital of Nanchang University, Medical Graduate School, Nanchang University, Nanchang, China; 2 Department of Thoracic Surgery, School of Medicine, Shanghai Pulmonary Hospital, Tongji University, 507 Zhengmin Road, Shanghai, 200433 China; 3 Department of General Surgery, School of Medicine, Shanghai Pulmonary Hospital, Tongji University, 507 Zhengmin Road, Shanghai, 200433 China

**Keywords:** Sublobectomy, Lobectomy, Pneumonectomy, Small cell lung cancer, SEER

## Abstract

**Purpose:**

The NCCN guidelines do not recommend surgery for T3–4N0M0/T1–4N1–2M0 small cell lung cancer (SCLC) due to a lack of evidence.

**Methods:**

Data of patients with T3–4N0M0/T1–4N1–2M0 SCLC were extracted from the Surveillance, Epidemiology, and End Results (SEER) database to determine the impact of surgery on this population. The Kaplan–Meier method, univariable and multivariable Cox proportional hazard regression, and propensity score matching (PSM) were used to compare the overall survival (OS) between the surgery and non-surgery groups. In addition, we explored whether sublobectomy, lobectomy, and pneumonectomy could provide survival benefits.

**Results:**

In total, 8572 patients with SCLC treated without surgery and 342 patients treated with surgery were included in this study. The PSM-adjusted hazard ratio (HR, 95% CI) for surgery vs. no surgery, sublobectomy vs. no surgery, lobectomy vs. no surgery, pneumonectomy vs. no surgery, and lobectomy plus adjuvant chemoradiotherapy vs. chemoradiotherapy were 0.71 (0.61–0.82) (*P* < 0.001), 0.91 (0.70–1.19) (*P* = 0.488), 0.60 (0.50–0.73) (*P* < 0.001), 0.57 (0.28–1.16) (*P* = 0.124), and 0.73 (0.56–0.96) (*P* = 0.023), respectively. The subgroup analysis demonstrated consistent results.

**Conclusions:**

Lobectomy improved OS in patients with T3–4N0M0/T1–4N1–2M0 SCLC, while pneumonectomy also demonstrated a tendency to improve OS without statistical significance; however, sublobectomy showed no survival benefit.

**Supplementary Information:**

The online version contains supplementary material available at 10.1186/s12957-023-03196-2.

## Introduction

Lung cancer is the leading cause of cancer-specific death worldwide despite a decrease in its incidence rate from the first to second place, as reported in a recent study [1]. Small cell lung cancer (SCLC), the most aggressive histologic type of lung cancer, constitutes approximately 15% of all lung cancers and is well known for its rapid proliferation, early metastases, and poor survival outcome [2]. Patients with SCLC are usually diagnosed with lymph node involvement or distant metastases, and the 5-year overall survival (OS) rate is only approximately 5–10% [3–7]. The median OS for untreated SCLC patients is only 2–4 months, whereas that for treated patients is 16–24 months for limited-stage disease and 6–12 months for extensive-stage disease [8].

Most patients with SCLC respond to chemotherapy; however, the 2-year disease-free survival rate is only approximately 10%, indicating that most patients experience recurrence within a short time after first-line therapy [8, 9]. For patients with AJCC eighth edition I–IIA SCLC, surgical resection is recommended in the latest NCCN guidelines; however, for more advanced-stage SCLC, surgery is not recommended [10]. Only 10% of SCLC patients undergo surgical resection [11], and most studies on surgery for SCLC mainly concentrate on the population with limited-stage disease [12–16]. However, studies focusing on the surgical benefit for locally advanced diseases are rare. Some studies indicated that the optimal surgical type for limited-stage SCLC was lobectomy, demonstrating better survival outcome than no surgery, sublobectomy, or pneumonectomy [17–19]; however, whether locally advanced SCLC could benefit from all the different surgical types remains inconclusive.

To explore the impact of surgery on locally advanced SCLC, we extracted data of patients with stage T3–4N0M0 and T1–4N1–2M0 SCLC from the Surveillance, Epidemiology, and End Results (SEER) database and performed the present study. In addition, the OS between the sublobectomy vs. no surgery, lobectomy vs. no surgery, and pneumonectomy vs. no surgery groups were compared to determine the influence of the different surgical types on this population.

## Methods

### Study cohort

SCLC data was extracted from the SEER database, a National Cancer Institute program covering 28% of the population in US population (https://www.seer.cancer.gov). Histological subtypes were coded using the third version of the International Classification of Diseases for Oncology (ICD-O-3). SCLC denoted “Site recode ICD-O-3/WHO 2008” of “Lung and Bronchus” and “Histologic Type ICD-O-3” of “8041–8045.” Seventeen registries collected the cancer patient characteristics, including demographics, tumor features, treatment strategies, and follow-up information.

After modifying the 6th or 7th TNM staging system into the 8th edition, 9267 patients with SCLC (only one primary tumor) with AJCC TNM 8th edition stage T3–4N0M0 or T1–4N1–2M0 disease (NCCN-guideline-recommended non-surgical candidates) were screened from January 2004 to December 2015. The following patients were included in the final cohort: those who (I) were treated with no surgery, sublobectomy, lobectomy, or pneumonectomy; (II) were diagnosed with pathological confirmation of SCLC; (III) were not treated with neoadjuvant/intraoperative radiotherapy/systemic therapy; and (IV) had known information about potential prognostic factors. All data in the SEER database was de-identified, and no patient had the risk of personal information leakage; therefore, the requirement of informed consent of the patients and ethical approval was exempted from the institutional review board of Medical Graduate School, Nanchang University. This study was conducted in accordance with the Declaration of Helsinki (as revised in 2013) and the Harmonized Tripartite Guideline for Good Clinical Practice from the International Conference on Harmonization.

### Covariables and endpoint

Baseline demographic and medical characteristics were extracted from the SEER database. The covariables included the year of diagnosis, age at diagnosis, gender, race, marital status at diagnosis, laterality, primary site, histological subtype, differentiation, TNM stage, radiotherapy, and chemotherapy. The year of diagnosis was categorized into 2004–2009 and 2010–2015, and the age at diagnosis was grouped into < 65 years and ≥ 65 years. The endpoint was OS which is defined as the time (months) from SCLC diagnosis to all-cause death. The most recent follow-up period was till December 2019.

### Statistical analysis

Categorical variables are shown as counts (percentages) and baseline characteristics of patients who underwent surgery and those who did not were compared using Pearson’s chi-square test or Fisher’s exact test as appropriate. Kaplan–Meier curves were used to demonstrate the time-dependent survival rate and were compared using a two-sided log-rank test. All potential prognostic factors were analyzed using the univariable Cox proportional hazards regression model (Cox regression), and those with *P* < 0.1 were then entered into the multivariable Cox model for further analysis. To further diminish the imbalance of baseline features between no surgery and surgery groups, the propensity score was calculated using a Logistic regression model and propensity score matching (PSM) with a caliper of 0.2, and a ratio of 1:2 was used to create matched cohorts regarding surgery vs. no surgery, sublobectomy vs. no surgery, lobectomy vs. no surgery, pneumonectomy vs. no surgery, and lobectomy plus adjuvant chemoradiotherapy vs. chemoradiotherapy. The same PSM strategy was also applied for surgery vs. no surgery in different TNM stage diseases (stages IIB, IIIA, and IIIB). The variables year of diagnosis, age at diagnosis, gender, race, marital status at diagnosis, laterality, primary site, histological subtype, differentiation, TNM stage, radiotherapy, and chemotherapy were entered into the Logistic model for propensity score calculation. A standardized mean difference (SMD) smaller than 0.1 between the two groups was considered a good balance.

The statistical analyses were conducted utilizing R software (http://www.r-project.org). All tests were two-sided, and a *P* value < 0.05 was deemed as statistical significance.

## Results

### Baseline characteristics

**Fig. 1:**
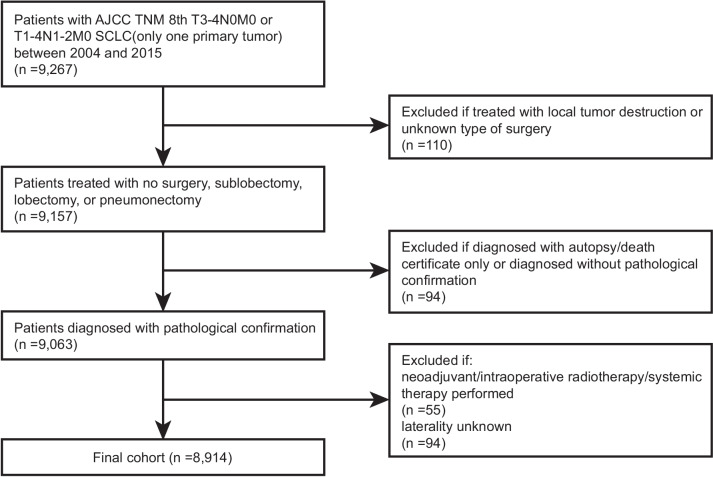
Flowchart of selection of study cohort

From January 2004 to December 2015, the SEER database included 9267 patients with SCLC (only one primary tumor) and AJCC TNM 8th edition stage T3–4N0M0 or T1–4N1–2M0 disease (stage IIB–IIIB). As shown in Fig. 1, after applying the screening criteria, 8914 patients (8572 who underwent no surgery and 342 who underwent surgery) were included in the final cohort. Baseline characteristics are listed in Table 1. Patients who underwent surgery were more likely to be married, have left-sided or lower lobe disease, have histology of combined small cell carcinoma, have an earlier TNM stage, and receive no radiotherapy or chemotherapy compared with those who did not undergo surgery. After PSM, all baseline characteristics showed an SMD smaller than or very close to 0.1 between the surgery and no surgery groups (Supplementary Fig. 1A).

**Table 1:** Baseline characteristics of stage T3–4N0M0/T1–4N1–2M0 SCLC patients stratified by surgical treatment

Variables	No surgery *n* = 8572	Surgery *n* = 342	*P*
**Year of diagnosis**			0.198
2004–2009	4132 (48.20%)	177 (51.75%)	
2010–2015	4440 (51.80%)	165 (48.25%)	
**Age**			0.794
< 65 years old	3595 (41.94%)	141 (41.23%)	
≥ 65 years old	4977 (58.06%)	201 (58.77%)	
**Gender**			0.801
Male	3976 (46.38%)	161 (47.08%)	
Female	4596 (53.62%)	181 (52.92%)	
**Race**			0.195
White	7375 (86.04%)	306 (89.47%)	
Black	790 (9.22%)	24 (7.02%)	
Other	407 (4.75%)	12 (3.51%)	
**Marital status**			0.01
Single	1083 (12.63%)	31 (9.06%)	
Married	4223 (49.27%)	199 (58.19%)	
Divorced/widowed/separated	2955 (34.47%)	100 (29.24%)	
Unknown	311 (3.63%)	12 (3.51%)	
**Laterality**			< 0.001
Right	5136 (59.92%)	174 (50.88%)	
Left	3436 (40.08%)	168 (49.12%)	
**Primary site**			< 0.001
Upper lobe	4642 (54.15%)	193 (56.43%)	
Middle lobe	392 (4.57%)	19 (5.56%)	
Lower lobe	1725 (20.12%)	112 (32.75%)	
Other	1813 (21.15%)	18 (5.26%)	
**Combined small cell carcinoma**			< 0.001
No/unknown	8445 (98.52%)	277 (80.99%)	
Yes	127 (1.48%)	65 (19.01%)	
**Differentiation**			< 0.001*
Grade I/II	36 (0.42%)	12 (3.51%)	
Grade III	862 (10.06%)	113 (33.04%)	
Grade IV	1752 (20.44%)	111 (32.46%)	
Unknown	5922 (69.09%)	106 (30.99%)	
**TNM stage**			< 0.001
IIB	890 (10.38%)	167 (48.83%)	
IIIA	3883 (45.30%)	139 (40.64%)	
IIIB	3799 (44.32%)	36 (10.53%)	
**Radiotherapy**			< 0.001
No/unknown	2910 (33.95%)	173 (50.58%)	
Yes	5662 (66.05%)	169 (49.42%)	
**Chemotherapy**			0.012
No/unknown	1749 (20.40%)	89 (26.02%)	
Yes	6823 (79.60%)	253 (73.98%)	

Categorical variables are presented with number (percentage)

*SCLC* small cell lung cancer, *PSM* propensity score matching

^*^P value was calculated with Fisher’s exact test

### Survival analysis

With follow-up until December 2019 and a median follow-up time (interquartile range, [IQR]) of 12 (5–26) months, 7927 patients in the no surgery group (*n* = 8572) and 289 in the surgery group (*n* = 342) died. The median OS (95% confidence interval, 95% CI) for the no surgery group and surgery groups was 12 (12–13) months and 20 (17–22) months, respectively. The 1-, 3-, and 5-year OS rates (95% CI) for the no surgery group were 49.36% (48.31–50.43%), 17.18% (16.40–18.00%), and 11.15% (10.50–11.85%), respectively. For the surgery group, the corresponding rates were 69.21% (64.48–74.29%), 29.50% (25.03–34.77%), and 20.96% (17.02–25.82%), respectively. The median OS (95% CI) for the no surgery and surgery groups after PSM was 13 (11–14) months and 19 (17–22) months, respectively.

**Fig. 2:**
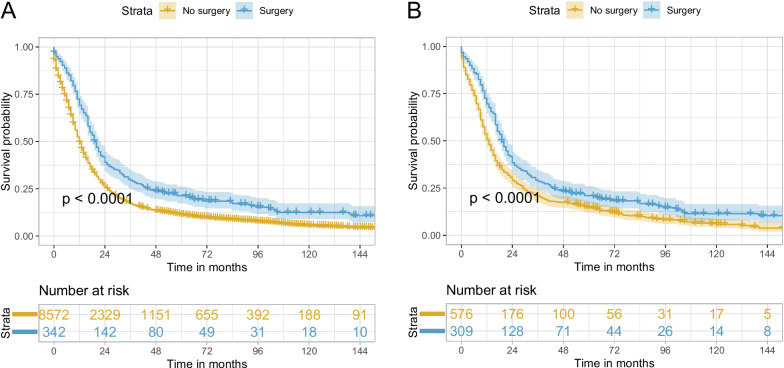
Kaplan–Meier curves of OS for stage T3–4N0M0/T1–4N1–2M0 SCLC comparing surgery with no surgery before PSM (**A**) and after PSM (**B**). OS, overall survival; SCLC, small cell lung cancer, PSM, propensity score matching

Table 2 and Supplementary Table 1 showed the hazard ratio (HR) (95% CI) for surgery vs. no surgery was 0.54 (0.48–0.61) (*P* < 0.001) in multivariable Cox regression analysis. The PSM-adjusted HR (95% CI) for surgery vs. no surgery was 0.71 (0.61–0.82) (*P* < 0.001). Overall, surgery, year of diagnosis, age at diagnosis, gender, marital status at diagnosis, primary site, TNM stage, radiotherapy, and chemotherapy were independent prognostic factors for OS in patients with locally advanced SCLC. The Kaplan–Meier curves indicated better OS in the surgery group than in the no surgery group both before and after PSM (both log-rank tests *P* < 0.001, Fig. 2).

**Table 2:** Univariable/multivariable Cox regression analysis and PSM-adjusted Cox regression analysis of the influence of surgery on OS of stage T3–4N0M0/T1–4N1–2M0 SCLC

Comparison	Before PSM				After PSM	
	**Univariable analysis**		**Multivariable analysis**			
	**HR (95% CI)**	***P***	**HR (95% CI)**	***P***	**HR (95% CI)**	***P***
**Surgery vs. no surgery**	0.66 (0.59–0.74)	< 0.001	0.54 (0.48–0.61)	< 0.001	0.71 (0.61–0.82)	< 0.001
**Sublobectomy vs. no surgery**	0.88 (0.71–1.08)	0.223	0.69 (0.56–0.85)	< 0.001	0.91 (0.70–1.19)	0.488
**Lobectomy vs. no surgery**	0.58 (0.50–0.67)	< 0.001	0.48 (0.41–0.56)	< 0.001	0.60 (0.50–0.73)	< 0.001
**Pneumonectomy vs. no surgery**	0.73 (0.44–1.19)	0.201	0.63 (0.39–1.03)	0.068	0.57 (0.28–1.16)	0.124
**Lobectomy plus chemoradiotherapy vs. chemoradiotherapy**	0.66 (0.54–0.82)	< 0.001	0.76 (0.61–0.94)	0.010	0.73 (0.56–0.96)	0.023

*PSM* propensity score matching, *OS* overall survival, *SCLC* small cell lung cancer, *HR* hazard ratio, *CI*, confidential interval

### Subgroup and sensitivity analysis

**Fig. 3:**
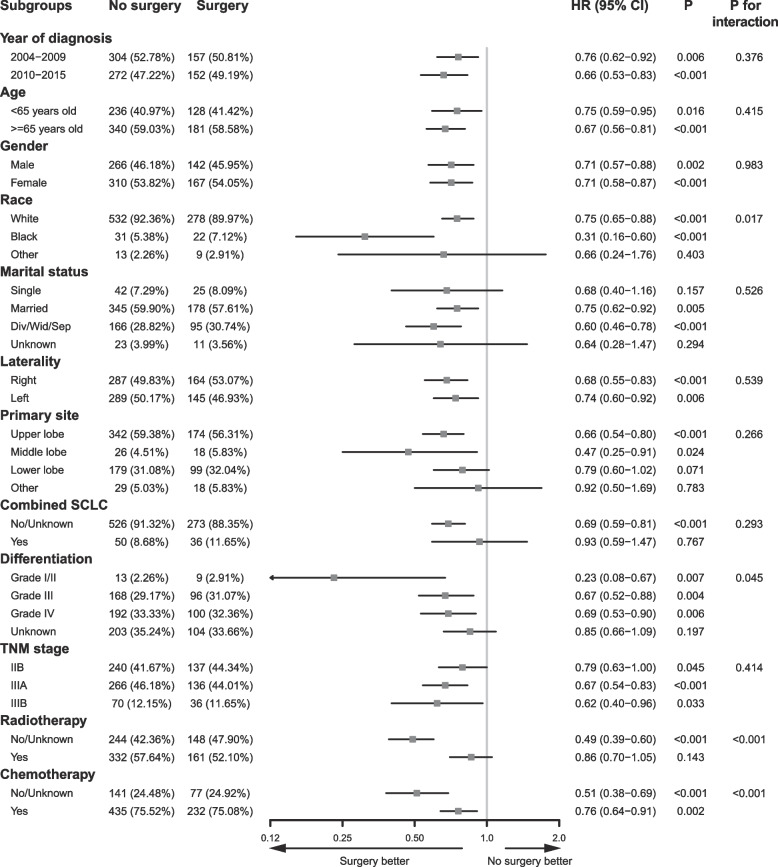
Forest plot for HR of surgery vs. no surgery in OS of stage T3–4N0M0/T1–4N1–2M0 SCLC after PSM. HR, hazard ratio; OS, overall survival; SCLC, small cell lung cancer; CI, confidential interval; PSM, propensity score matching

A subgroup analysis was performed on the PSM cohort using univariable Cox regression analysis. The results showed a consistent tendency favoring surgery, although some subgroups did not achieve statistical significance (Fig. 3).

**Fig. 4:**
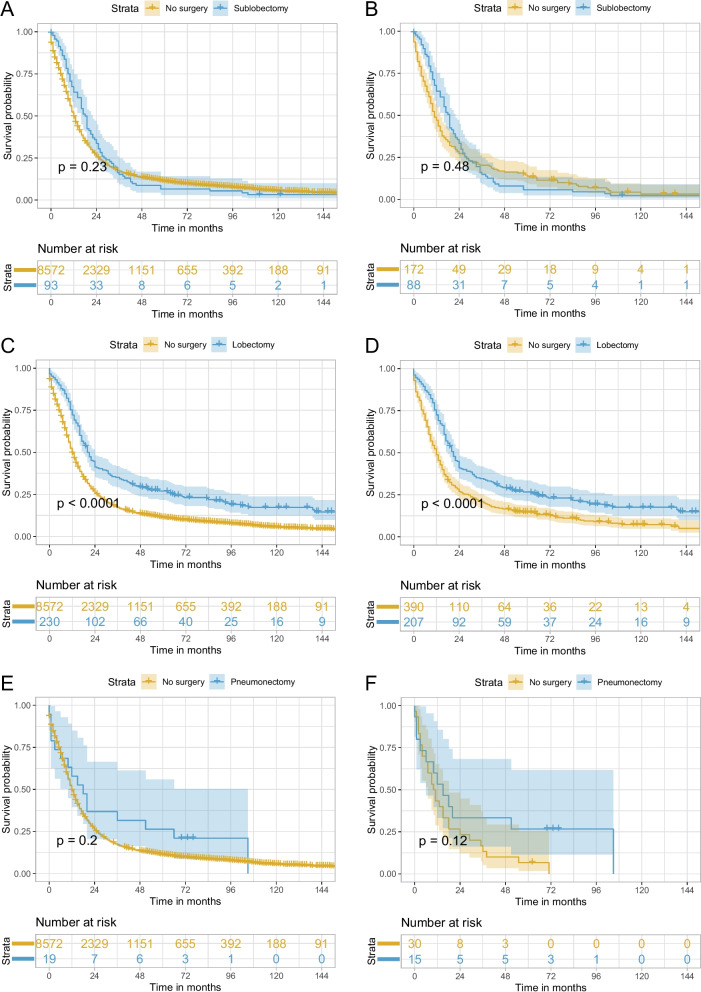
Kaplan–Meier curves of OS for stage T3–4N0M0/T1–4N1–2M0 SCLC for sublobectomy vs. no surgery before PSM (**A**) and after PSM (**B**), lobectomy vs. no surgery before PSM (**C**) and after PSM (**D**), and pneumonectomy vs. no surgery before PSM (**E**) and after PSM (**F**). OS, overall survival; SCLC, small cell lung cancer, PSM, propensity score matching

**Fig. 5:**
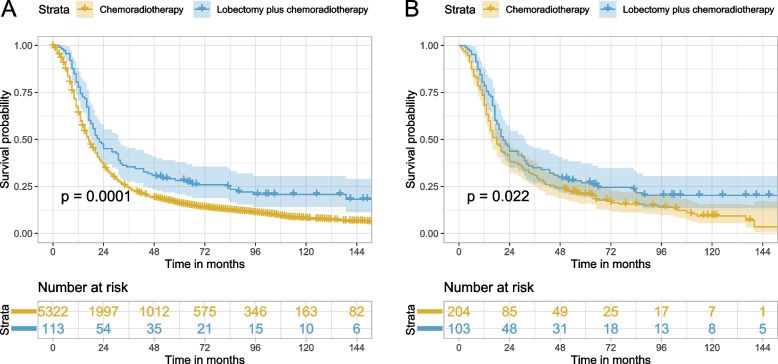
Kaplan–Meier curves of OS for stage T3–4N0M0/T1–4N1–2M0 SCLC comparing lobectomy plus chemoradiotherapy with chemoradiotherapy before PSM (**A**) and after PSM (**B**). OS, overall survival; SCLC, small cell lung cancer, PSM, propensity score matching

**Fig. 6:**
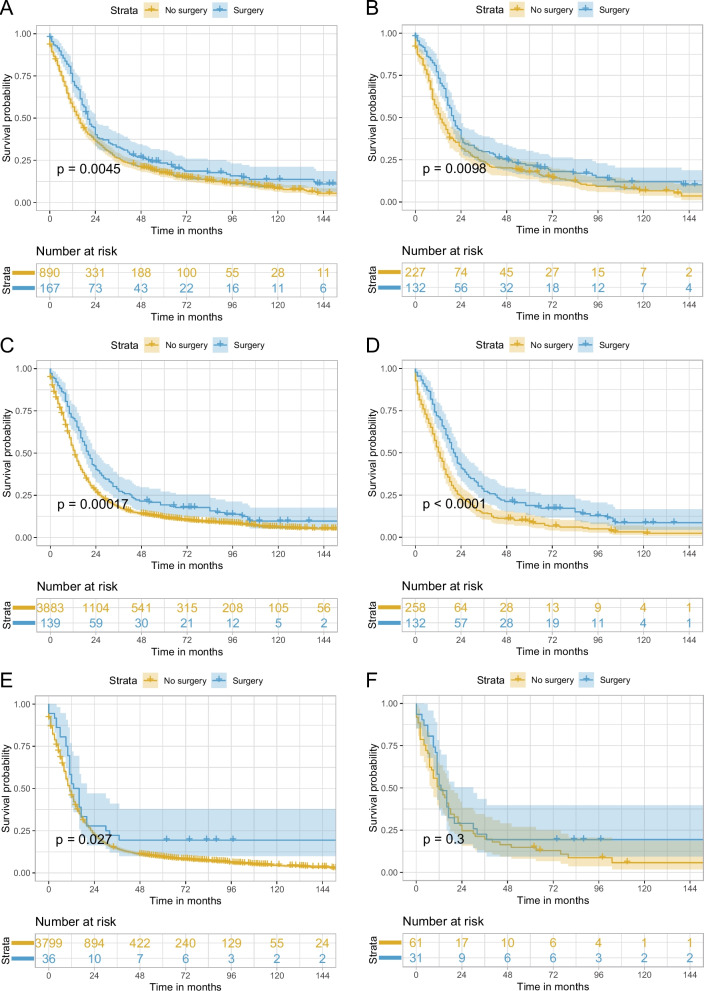
Kaplan–Meier curves of OS for surgery vs. no surgery for SCLC patients with stage IIB disease before PSM (**A**) and after PSM (**B**), IIIA disease before PSM (**C**) and after PSM (**D**), and IIIB disease before PSM (**E**) and after PSM (**F**). OS, overall survival; SCLC, small cell lung cancer, PSM, propensity score matching

In the sensitivity analysis, variable surgery was categorized into no surgery, sublobectomy, lobectomy, and pneumonectomy to detect the impact of different surgical types. In addition, the effects of lobectomy plus adjuvant chemoradiotherapy and chemoradiotherapy were also compared. PSM was also applied to all the comparisons between the different surgical types and no surgery. After PSM, the baseline characteristics of the surgery and no surgery group showed a good balance (Supplementary Fig. 1B–E). As demonstrated in Table 2 and Supplementary Table 2, the HR (95% CI) for multivariable Cox regression analysis for sublobectomy vs. no surgery, lobectomy vs. no surgery, pneumonectomy vs. no surgery, and lobectomy plus adjuvant chemoradiotherapy vs. chemoradiotherapy were 0.69 (0.56–0.85) (*P* < 0.001), 0.48 (0.41–0.56) (*P* < 0.001), 0.63 (0.39–1.03) (*P* = 0.068), and 0.76 (0.61–0.94) (*P* = 0.010), respectively, showing significant OS benefit for sublobectomy/lobectomy and an almost significant OS benefit for pneumonectomy. The corresponding results in PSM-adjusted analysis were 0.91 (0.70–1.19) (*P* = 0.488), 0.60 (0.50–0.73) (*P* < 0.001), 0.57 (0.28–1.16) (*P* = 0.124), and 0.73 (0.56–0.96) (*P* = 0.023), respectively, showing significant OS benefit for lobectomy and a tendency toward OS benefit for pneumonectomy, while the point estimate of HR for sublobectomy vs. no surgery was close to 1. Kaplan–Meier curves in Figs. 4 and 5 showed similar results to Table 2 and Supplementary Table 2: lobectomy showed significantly better OS (log-rank test *P* < 0.001 before and after PSM), and sublobectomy (log-rank test *P* = 0.230 before PSM and *P* = 0.480 after PSM) and pneumonectomy (log-rank test *P* = 0.200 before PSM and *P* = 0.120 after PSM) presented no significant OS difference compared with no surgery. Lobectomy plus adjuvant chemoradiotherapy exhibited better OS than chemoradiotherapy (log-rank test *P* < 0.001 before PSM and *P* = 0.022 after PSM). Further investigation of the impact of surgery on different TNM stage disease was performed. PSM was also applied to different TNM stage disease and the baseline characteristics after PSM for surgery vs. no surgery for stages IIB, IIIA, and IIIB were well balanced (Supplementary Fig. 2A–C). In Table 3, HR (95% CI) for multivariable Cox regression analysis of surgery vs. no surgery in stage IIB, IIIA, and IIIB disease were 0.57 (0.46–0.70) (*P* < 0.001), 0.53 (0.44–0.64) (*P* < 0.001), and 0.58 (0.40–0.84) (*P* = 0.004), and the PSM-adjusted HR (95% CI) for surgery vs. no surgery in stages IIB and IIIA showed similar results: 0.74 (0.59–0.93) (*P* = 0.010) and 0.61 (0.49–0.77) (*P* < 0.001), while the corresponding data in stage IIIB showed a tendency favoring surgery but without statistical significance: 0.78 (0.49–1.26) (*P* = 0.311). Log-rank test and Kaplan–Meier curves in Fig. 6 exhibited similar results, showing that only stage IIB and IIIA disease achieved significant OS benefit after PSM. What is more, sensitivity analyses adjusting tumor size, T classification, and N classification were also performed and demonstrated similar results to the main analyses (Supplementary Fig. 3–4). In the subgroups of patients with stage IIIB and IIIC disease, different surgical types were compared with no surgery after PSM, respectively, and only lobectomy in patients with stage IIIA disease could improve OS (Supplementary Fig. 5–8). Pneumonectomy was not analyzed in patients with IIIB disease due to only two patients underwent pneumonectomy in this population.

**Table 3:** Sensitivity analysis with univariable/multivariable Cox regression and PSM-adjusted Cox regression of the HR for surgery vs. no surgery for OS of stage IIB, IIIA, and IIIB SCLC

TNM stage	Before PSM				After PSM	
	**Univariable analysis**		**Multivariable analysis**			
	**HR (95% CI)**	***P***	**HR (95% CI)**	***P***	**HR (95% CI)**	***P***
**IIB–IIIB**	0.66 (0.59–0.74)	< 0.001	0.54 (0.48–0.61)	< 0.001	0.71 (0.61–0.82)	< 0.001
**IIB**	0.77 (0.64–0.92)	0.005	0.57 (0.46–0.70)	< 0.001	0.74 (0.59–0.93)	0.010
**IIIA**	0.71 (0.59–0.85)	< 0.001	0.53 (0.44–0.64)	< 0.001	0.61 (0.49–0.77)	< 0.001
**IIIB**	0.67 (0.46–0.96)	0.030	0.58 (0.40–0.84)	0.004	0.78 (0.49–1.26)	0.311

*PSM* propensity score matching, *OS* overall survival, *SCLC* small cell lung cancer, *HR* hazard ratio, *CI* confidential interval

## Discussion

SEER database is a good source for investigating those issues for which it is difficult to collect large samples in clinical practice, such as the role of surgery in locally advanced SCLC. However, the SEER database includes different editions of TNM staging systems. To provide directly applicable evidence for the present clinical treatment, we modified the sixth and seventh AJCC TNM staging system into the eighth version.

In the present multicenter population-based study, only 3.8% (*n* = 342) of the entire study cohort (*n* = 8914) underwent surgery, suggesting the inclination of clinicians to provide conservative treatment. Univariable Cox regression and Kaplan–Meier curves showed that the surgery group exhibited better OS than the no surgery group; however, sublobectomy and pneumonectomy showed no significant differences compared with no surgery, and only lobectomy was superior to no surgery. In addition, univariable analyses demonstrated results in favor of surgery in all patients with stage IIB–IIIB SCLC. Multivariable analyses showed similar results, except for the sensitivity analysis in patients with stage IIIB disease. Using multivariable Cox regression and PSM-adjusted Cox regression, we found that surgery could improve OS in patients with stage IIB–IIIB (T3–4N0M0 and T1–4N1–2) SCLC. All subgroups of the PSM cohort tended to favor surgery. In the sensitivity analysis, among the three surgical types, only lobectomy significantly enhanced the OS outcome in this population after PSM. In addition, a statistically significant survival benefit with surgery was observed in stage IIB and IIIA SCLC only, and not in IIIB SCLC after PSM. To be noted, the HR (95% CI) for pneumonectomy vs. no surgery in the entire study cohort after PSM was 0.57 (0.28–1.16) (Table 2), and the HR (95% CI) for surgery vs. no surgery in stage IIIB cohort after PSM was 0.78 (0.49–1.26) (Table 3), showing a tendency favoring pneumonectomy in the whole study cohort and surgery in the stage IIIB cohort although without statistical significance. The sample sizes of the pneumonectomy group (*n* = 19) in the entire study cohort (Fig. 4) and the surgery group (*n* = 36) in the stage IIIB cohort (Fig. 5) were very small which may explain why there was no significant difference.

The impact of surgery on SCLC has been measured repeatedly. Before the 1970s, surgery was often performed for SCLC. A Medical Research Council trial conducted in 1973 comparing surgery with radiotherapy showed worse survival in the surgery group [20]; subsequently, surgery was no longer recommended. This study had several limitations: first, no stage information of the SCLC was reported; second, only pneumonectomy was performed; third, only 48% of the surgery group underwent radical resection, 38% underwent open and close, and 18% did not undergo surgery. An RCT study conducted by Lad et al. randomized 146 limited stage SCLC patients who responded to chemotherapy into groups with and without surgery, and no significant difference in OS was observed (median OS for surgery vs. no surgery: 15.4 vs. 18.6 months, *P* = 0.78). This study was well-designed except that the sample size was small, and subgroup analysis relating to the surgical type and stage was not reported. In our study, 8914 patients with locally advanced SCLC were included in the analysis; even after PSM, there were still 309 and 576 patients in the surgery and no surgery groups, respectively; and the median OS for surgery vs. no surgery in the PSM cohort was 19 vs. 13 months, indicating an improvement of half a year on OS of patients with locally advanced SCLC.

The role of different surgical types in limited-stage or resectable SCLC have been reported in several retrospective studies [17–19]. These three studies suggested that surgery was superior to no surgery and that lobectomy conferred better survival than sublobar resection or pneumonectomy. In a study focusing on stage III SCLC conducted by Gao et al., surgery also improved OS, and lobectomy resulted in better OS than sublobectomy or pneumonectomy; however, comparison between sublobectomy vs. no surgery, lobectomy vs. no surgery, and pneumonectomy vs. no surgery were not mentioned. In our study, sublobectomy showed no significant survival benefit with a large sample size (Fig. 4A and B), while pneumonectomy showed a tendency towards OS improvement; however, no significant difference was observed, which might be due to a small sample size (Fig. 4E and F). Therefore, further studies relating to pneumonectomy in locally advanced SCLC are needed in the future. As few surgeons would recommend pneumonectomy for this special population, it is difficult to enroll patients in a prospective randomized controlled trial. A retrospective study might be suitable to illustrate the role of pneumonectomy. Currently, systemic therapy and radiotherapy are recommended for patients with SCLC with IIB and higher stage in the recent NCCN guidelines [10], and whether adding lobectomy to these treatments could result in better OS is unknown. A retrospective study conducted by Wakeam et al. reported that lobectomy and adjuvant chemotherapy were associated with significantly longer OS comparing with chemoradiotherapy for stage I/II SCLC [21]. Ning et al. reported that adding lobectomy but not sublobectomy to chemoradiotherapy could improve OS for T1–2N0M0 SCLC patients aged ≥ 65 years [13]. Similar result was observed in our research of locally advanced SCLC patients with PSM-adjusted HR (95% CI) of 0.73 (0.56–0.96) (*P* = 0.023) for lobectomy plus adjuvant chemoradiotherapy vs. chemoradiotherapy. Although radiotherapy seemed to reduce the degree of benefit from surgery as shown in Fig. 3, lobectomy plus adjuvant chemoradiotherapy was superior to chemoradiotherapy alone in the study cohort (Table 2 and Fig. 5). Overall, lobectomy is a promising treatment, sublobectomy is not recommended, and the impact of pneumonectomy is still obscure for patients with stage IIB–IIIB SCLC.

Whether surgery can enhance the prognosis for all stage IIB, IIIA, and IIIB SCLC is also unclear. Gao et al. reported a better prognosis of surgery than no surgery in the subgroup of IIIA, IIIB, and IIIC in the post-PSM cohort [22]. These results are consistent with the significant results in subgroups of stage IIIA and IIIB in the post-PSM cohort demonstrated in Fig. 3. Sensitivity analysis of the stage IIIB cohort also showed a similar tendency although without statistical significance which may be related to the small sample size (Fig. 6E and F). To be noted, the study cohort in Gao et al.’s and our study differed in that patients with neoadjuvant therapy were excluded in our study which was not the case in Gao et al.’s, indicating that our study demonstrated that even without neoadjuvant therapy, stage IIB–IIIA SCLC patients can benefit from surgery, and stage IIIB SCLC patients can potentially benefit from surgery which should be validated with more samples.

The breakthroughs in resectable non-small cell lung cancers (NSCLCs) brought by immune checkpoint inhibitors (ICIs) in the setting of neoadjuvant or adjuvant therapy have been reported in some studies [23–25]. Neoadjuvant chemoimmunotherapy can transform the unresectable NSCLCs into resectable tumors [26]. Some RCTs have shown promising results for extensive-stage SCLC [27]. These studies provide prospects for downstaging unresectable locally advanced SCLC via neoadjuvant immunotherapy combined with chemotherapy or other systemic treatments to increase the chance of resection. As many new ICIs are available in clinical practice, RCTs are needed to explore the role of surgery in combination with neoadjuvant/adjuvant therapy in locally advanced SCLC. As the surgery is not recommended by the current guidelines for stage IIB–IIIB SCLC, the choice of surgery and perioperative therapy in this population should be made prudently after the discussion of the potential benefits and risks by the multidiscipline team.

Some limitations of the present research have to be noted. First, there may have been some inherent bias due to the retrospective nature of the study. Second, some baseline variables such as smoking status, cardiopulmonary function, detailed usage, and chemotherapy/radiotherapy dosage, which could influence the endpoint, were not recorded in the SEER database.

## Conclusion

Lobectomy improved the OS in patients with T3–4N0M0/T1–4N1–2M0 SCLC. Pneumonectomy also showed a tendency to improve the OS without statistical significance; however, sublobectomy showed no survival benefit.

### Supplementary Information

**Additional file 1: Supplementary Table 1.** Univariable/multivariable Cox regression analysis of the influence of surgery (surgery vs. no surgery) on OS of stage T3-4N0M0/T1-4N1-2M0 SCLC. **Supplementary Table 2.** Univariable/multivariable Cox regression analysis of the influence of surgery (sublobectomy/lobectomy/pneumonectomy vs. no surgery) on OS of stage T3-4N0M0/T1-4N1-2M0 SCLC. **Supplementary Fig. 1.** SMD between surgery and no surgery (A), sublobectomy and no surgery (B), lobectomy and no surgery (C), pneumonectomy and no surgery (D) before and after PSM, lobectomy plus chemoradiotherapy and chemoradiotherapy (E) before and after PSM. The dotted lines denote the SMD of -0.1 and 0.1. SMD, standardized mean difference; PSM, propensity score matching. **Supplementary Fig. 2.** SMD between surgery and no surgery in patients with stage IIB (A), stage IIIA (B), and stage IIIB (C) before and after PSM. The dotted lines denote the SMD of -0.1 and 0.1. SMD, standardized mean difference; PSM, propensity score matching. **Supplementary Fig. 3.** SMD between surgery vs. no surgery (A), sublobectomy vs. no surgery (B), lobectomy vs. no surgery (C), and pneumonectomy vs. no surgery (D) in patients with stage T3-4N0M0/T1-4N1-2M0 SCLC before and after PSM adjusting tumor size, T classification, N classification and other baseline characteristics. The dotted lines denote the SMD of -0.1 and 0.1. SMD, standardized mean difference; PSM, propensity score matching. **Supplementary Fig. 4.** Kaplan-Meier curves of OS for stage T3-4N0M0/T1-4N1-2M0 SCLC comparing surgery vs. no surgery (A), sublobectomy vs. no surgery (B), lobectomy vs. no surgery (C), and pneumonectomy vs. no surgery (D) after PSM adjusting tumor size, T classification, N classification and other baseline characteristics. OS, overall survival; SCLC, small cell lung cancer, PSM, propensity score matching. **Supplementary Fig. 5.** SMD between sublobectomy vs. no surgery (A), lobectomy vs. no surgery (B), and pneumonectomy vs. no surgery (C) in patients with stage IIIA SCLC before and after PSM. The dotted lines denote the SMD of -0.1 and 0.1. SMD, standardized mean difference; PSM, propensity score matching. **Supplementary Fig. 6. **Kaplan-Meier curves of OS for stage IIIA SCLC comparing sublobectomy vs. no surgery (A, B), lobectomy vs. no surgery (C, D), and pneumonectomy vs. no surgery (E, F) before and after PSM. OS, overall survival; SCLC, small cell lung cancer, PSM, propensity score matching. **Supplementary Fig. 7.** SMD between sublobectomy vs. no surgery (A) and lobectomy vs. no surgery (B) in patients with stage IIIB SCLC before and after PSM. The dotted lines denote the SMD of -0.1 and 0.1. SMD, standardized mean difference; PSM, propensity score matching. **Supplementary Fig. 8. **Kaplan-Meier curves of OS for stage IIIB SCLC comparing sublobectomy vs. no surgery (A, B) and lobectomy vs. no surgery (C, D) before and after PSM. OS, overall survival; SCLC, small cell lung cancer, PSM, propensity score matching.

## Data Availability

The dataset supporting the conclusions of this article is available in the SEER*Stat software (https://seer.cancer.gov/resources/).

## Abbreviations

AJCC: American Joint Committee on Cancer
CI: Confidence interval
HR: Hazard ratio
ICD-O-3: Third edition of the International Classification of Diseases for Oncology
ICIs: Immune checkpoint inhibitors
IQR: Interquartile range
NCCN: National Comprehensive Cancer Network
OS: Overall survival
PSM: Propensity score matching
SCLC: Small cell lung cancer
SEER: Surveillance, Epidemiology, and End Results
SMD: Standardized mean difference
TNM: Tumor, node, metastasis
