# Supplementary material for: Surgery for stage IIB–IIIB small cell lung cancer
Source: World J Surg Oncol. 2023 Oct 23;21:333. doi: 10.1186/s12957-023-03196-2 (PMC10591395; doi:10.1186/s12957-023-03196-2)
Supplement: Supplementary file 1 — Additional file 1: Supplementary Table 1. Univariable/multivariable Cox regression analysis of the influence of surgery (surgery vs. no surgery) on OS of stage T3-4N0M0/T1-4N1-2M0 SCLC. Supplementary Table 2. Univariable/multivariable Cox regression analysis of the influence of surgery (sublobectomy/lobectomy/pneumonectomy vs. no surgery) on OS of stage T3-4N0M0/T1-4N1-2M0 SCLC. Supplementary Fig. 1. SMD between surgery and no surgery (A), sublobectomy and no surgery (B), lobectomy and no surgery (C), pneumonectomy and no surgery (D) before and after PSM, lobectomy plus chemoradiotherapy and chemoradiotherapy (E) before and after PSM. The dotted lines denote the SMD of -0.1 and 0.1. SMD, standardized mean difference; PSM, propensity score matching. Supplementary Fig. 2. SMD between surgery and no surgery in patients with stage IIB (A), stage IIIA (B), and stage IIIB (C) before and after PSM. The dotted lines denote the SMD of -0.1 and 0.1. SMD, standardized mean difference; PSM, propensity score matching. Supplementary Fig. 3. SMD between surgery vs. no surgery (A), sublobectomy vs. no surgery (B), lobectomy vs. no surgery (C), and pneumonectomy vs. no surgery (D) in patients with stage T3-4N0M0/T1-4N1-2M0 SCLC before and after PSM adjusting tumor size, T classification, N classification and other baseline characteristics. The dotted lines denote the SMD of -0.1 and 0.1. SMD, standardized mean difference; PSM, propensity score matching. Supplementary Fig. 4. Kaplan-Meier curves of OS for stage T3-4N0M0/T1-4N1-2M0 SCLC comparing surgery vs. no surgery (A), sublobectomy vs. no surgery (B), lobectomy vs. no surgery (C), and pneumonectomy vs. no surgery (D) after PSM adjusting tumor size, T classification, N classification and other baseline characteristics. OS, overall survival; SCLC, small cell lung cancer, PSM, propensity score matching. Supplementary Fig. 5. SMD between sublobectomy vs. no surgery (A), lobectomy vs. no surgery (B), and pneumonectomy vs. no surgery ( [file 12957_2023_3196_MOESM1_ESM.docx]

**Supplementary Table 1** Univariable/multivariable Cox regression analysis of the influence of the influence of surgery (surgery vs. no surgery) on OS of stage T3-4N0M0/T1-4N1-2M0 SCLC

| **Variables** | **Univariable analysis** | | **Multivariable analysis** | |  |
| --- | --- | --- | --- | --- | --- |
|  | **HR (95% CI)** | **P** | **HR (95% CI)** | **P** |  |
| **Surgery** |  |  |  |  |  |
| No | 1 |  | 1 |  |  |
| Yes | 0.66 (0.59, 0.74) | <0.001 | 0.54 (0.48-0.61) | <0.001 |  |
| **Year of diagnosis** |  |  |  |  |  |
| 2004-2009 | 1 |  | 1 |  |  |
| 2010-2015 | 0.93 (0.89, 0.97) | 0.002 | 0.94 (0.90-0.98) | 0.008 |  |
| **Age** |  |  |  |  |  |
| <65 years old | 1 |  | 1 |  |  |
| >=65 years old | 1.57 (1.51, 1.65) | <0.001 | 1.41 (1.35-1.48) | <0.001 |  |
| **Gender** |  |  |  |  |  |
| Male | 1 |  | 1 |  |  |
| Female | 0.88 (0.85, 0.92) | <0.001 | 0.85 (0.81-0.89) | <0.001 |  |
| **Race** |  |  |  |  |  |
| White | 1 |  |  |  |  |
| Black | 0.98 (0.91, 1.05) | 0.534 |  |  |  |
| Other | 0.97 (0.88, 1.08) | 0.607 |  |  |  |
| **Marital status** |  |  |  |  |  |
| Single | 1 |  | 1 |  |  |
| Married | 0.99 (0.92, 1.06) | 0.678 | 0.99 (0.92-1.06) | 0.696 |  |
| Divorced/Widowed/Separated | 1.18 (1.10, 1.27) | <0.001 | 1.13 (1.05-1.22) | <0.001 |  |
| Unknown | 1.08 (0.94, 1.23) | 0.268 | 1.06 (0.93-1.21) | 0.372 |  |
| **Laterality** |  |  |  |  |  |
| Right | 1 |  |  |  |  |
| Left | 1.00 (0.95, 1.04) | 0.925 |  |  |  |
| **Primary site** |  |  |  |  |  |
| Upper lobe | 1 |  | 1 |  |  |
| Middle lobe | 1.08 (0.97, 1.20) | 0.144 | 1.04 (0.94-1.16) | 0.439 |  |
| Lower lobe | 1.11 (1.05, 1.17) | <0.001 | 1.11 (1.05-1.18) | <0.001 |  |
| Other | 1.10 (1.04, 1.16) | 0.001 | 1.07 (1.01-1.13) | 0.022 |  |
| **Combined small cell carcinoma** |  |  |  |  |  |
| No/Unknown | 1 |  |  |  |  |
| Yes | 0.96 (0.82, 1.11) | 0.547 |  |  |  |
| **Differentiation** |  |  |  |  |  |
| Grade I/II | 1 |  | 1 |  |  |
| Grade III | 1.30 (0.95, 1.78) | 0.096 | 1.23 (0.89-1.68) | 0.205 |  |
| Grade IV | 1.34 (0.99, 1.83) | 0.062 | 1.22 (0.90-1.67) | 0.206 |  |
| Unknown | 1.33 (0.98, 1.81) | 0.066 | 1.21 (0.89-1.65) | 0.223 |  |
| **TNM stage** |  |  |  |  |  |
| IIB | 1 |  | 1 |  |  |
| IIIA | 1.23 (1.14-1.32) | <0.001 | 1.20 (1.12-1.30) | <0.001 |  |
| IIIB | 1.43 (1.33-1.54) | <0.001 | 1.46 (1.35-1.57) | <0.001 |  |
| **Radiotherapy** |  |  |  |  |  |
| No/Unknown | 1 |  | 1 |  |  |
| Yes | 0.42 (0.40, 0.44) | <0.001 | 0.52 (0.49-0.54) | <0.001 |  |
| **Chemotherapy** |  |  |  |  |  |
| No/Unknown | 1 |  | 1 |  |  |
| Yes | 0.38 (0.36, 0.40) | <0.001 | 0.54 (0.51-0.58) | <0.001 |  |

SCLC, small cell lung cancer; OS, overall survival; HR, hazard ratio; CI, confidential interval.

**Supplementary Table 2** Univariable/multivariable Cox regression analysis of the influence of the influence of surgery (sublobectomy/lobectomy/pneumonectomy vs. no surgery) on OS of stage T3-4N0M0/T1-4N1-2M0 SCLC

| **Variables** | **Univariable analysis** | | **Multivariable analysis** | |  |
| --- | --- | --- | --- | --- | --- |
|  | **HR (95% CI)** | **P** | **HR (95% CI)** | **P** |  |
| **Surgery** |  |  |  |  |  |
| No | 1 |  | 1 |  |  |
| Sublobectomy | 0.88 (0.71, 1.08) | 0.223 | 0.69 (0.56-0.85) | <0.001 |  |
| Lobectomy | 0.58 (0.50, 0.67) | <0.001 | 0.48 (0.41-0.56) | <0.001 |  |
| Pneumonectomy | 0.73 (0.44, 1.19) | 0.201 | 0.63 (0.39-1.03) | 0.068 |  |
| **Year of diagnosis** |  |  |  |  |  |
| 2004-2009 | 1 |  | 1 |  |  |
| 2010-2015 | 0.93 (0.89, 0.97) | 0.002 | 0.94 (0.90-0.99) | 0.009 |  |
| **Age** |  |  |  |  |  |
| <65 years old | 1 |  | 1 |  |  |
| >=65 years old | 1.57 (1.51, 1.65) | <0.001 | 1.41 (1.35-1.48) | <0.001 |  |
| **Gender** |  |  |  |  |  |
| Male | 1 |  | 1 |  |  |
| Female | 0.88 (0.85, 0.92) | <0.001 | 0.85 (0.81-0.89) | <0.001 |  |
| **Race** |  |  |  |  |  |
| White | 1 |  |  |  |  |
| Black | 0.98 (0.91, 1.05) | 0.534 |  |  |  |
| Other | 0.97 (0.88, 1.08) | 0.607 |  |  |  |
| **Marital status** |  |  |  |  |  |
| Single | 1 |  | 1 |  |  |
| Married | 0.99 (0.92, 1.06) | 0.678 | 0.99 (0.92-1.06) | 0.693 |  |
| Divorced/Widowed/Separated | 1.18 (1.10, 1.27) | <0.001 | 1.13 (1.05-1.22) | <0.001 |  |
| Unknown | 1.08 (0.94, 1.23) | 0.268 | 1.07 (0.94-1.22) | 0.337 |  |
| **Laterality** |  |  |  |  |  |
| Right | 1 |  |  |  |  |
| Left | 1.00 (0.95, 1.04) | 0.925 |  |  |  |
| **Primary site** |  |  |  |  |  |
| Upper lobe | 1 |  | 1 |  |  |
| Middle lobe | 1.08 (0.97, 1.20) | 0.144 | 1.04 (0.94-1.16) | 0.446 |  |
| Lower lobe | 1.11 (1.05, 1.17) | <0.001 | 1.11 (1.05-1.18) | <0.001 |  |
| Other | 1.10 (1.04, 1.16) | 0.001 | 1.07 (1.01-1.13) | 0.026 |  |
| **Combined small cell carcinoma** |  |  |  |  |  |
| No/Unknown | 1 |  |  |  |  |
| Yes | 0.96 (0.82, 1.11) | 0.547 |  |  |  |
| **Differentiation** |  |  |  |  |  |
| Grade I/II | 1 |  | 1 |  |  |
| Grade III | 1.30 (0.95, 1.78) | 0.096 | 1.20 (0.88-1.65) | 0.25 |  |
| Grade IV | 1.34 (0.99, 1.83) | 0.062 | 1.20 (0.88-1.64) | 0.254 |  |
| Unknown | 1.33 (0.98, 1.81) | 0.066 | 1.19 (0.87-1.62) | 0.276 |  |
| **TNM stage** |  |  |  |  |  |
| IIB | 1 |  | 1 |  |  |
| IIIA | 1.23 (1.14-1.32) | <0.001 | 1.20 (1.11-1.29) | <0.001 |  |
| IIIB | 1.43 (1.33-1.54) | <0.001 | 1.45 (1.35-1.57) | <0.001 |  |
| **Radiotherapy** |  |  |  |  |  |
| No/Unknown | 1 |  | 1 |  |  |
| Yes | 0.42 (0.40, 0.44) | <0.001 | 0.52 (0.49-0.54) | <0.001 |  |
| **Chemotherapy** |  |  |  |  |  |
| No/Unknown | 1 |  | 1 |  |  |
| Yes | 0.38 (0.36, 0.40) | <0.001 | 0.54 (0.51-0.58) | <0.001 |  |

SCLC, small cell lung cancer; OS, overall survival; HR, hazard ratio; CI, confidential interval.

**
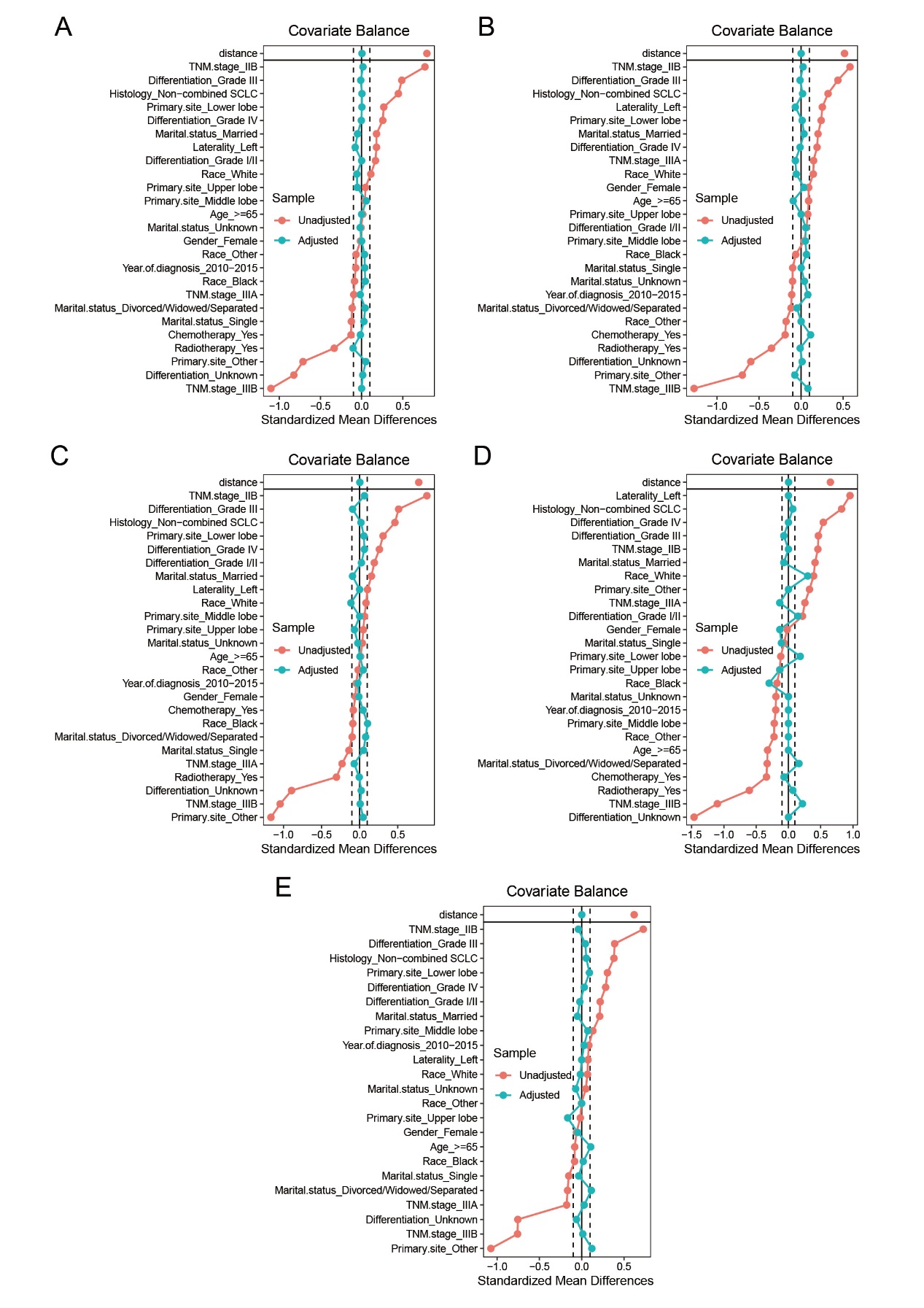
**

**Supplementary Fig. 1** SMD between surgery and no surgery (A), sublobectomy and no surgery (B), lobectomy and no surgery (C), pneumonectomy and no surgery (D) before and after PSM, lobectomy plus chemoradiotherapy and chemoradiotherapy (E) before and after PSM. The dotted lines denote the SMD of -0.1 and 0.1. SMD, standardized mean difference; PSM, propensity score matching.


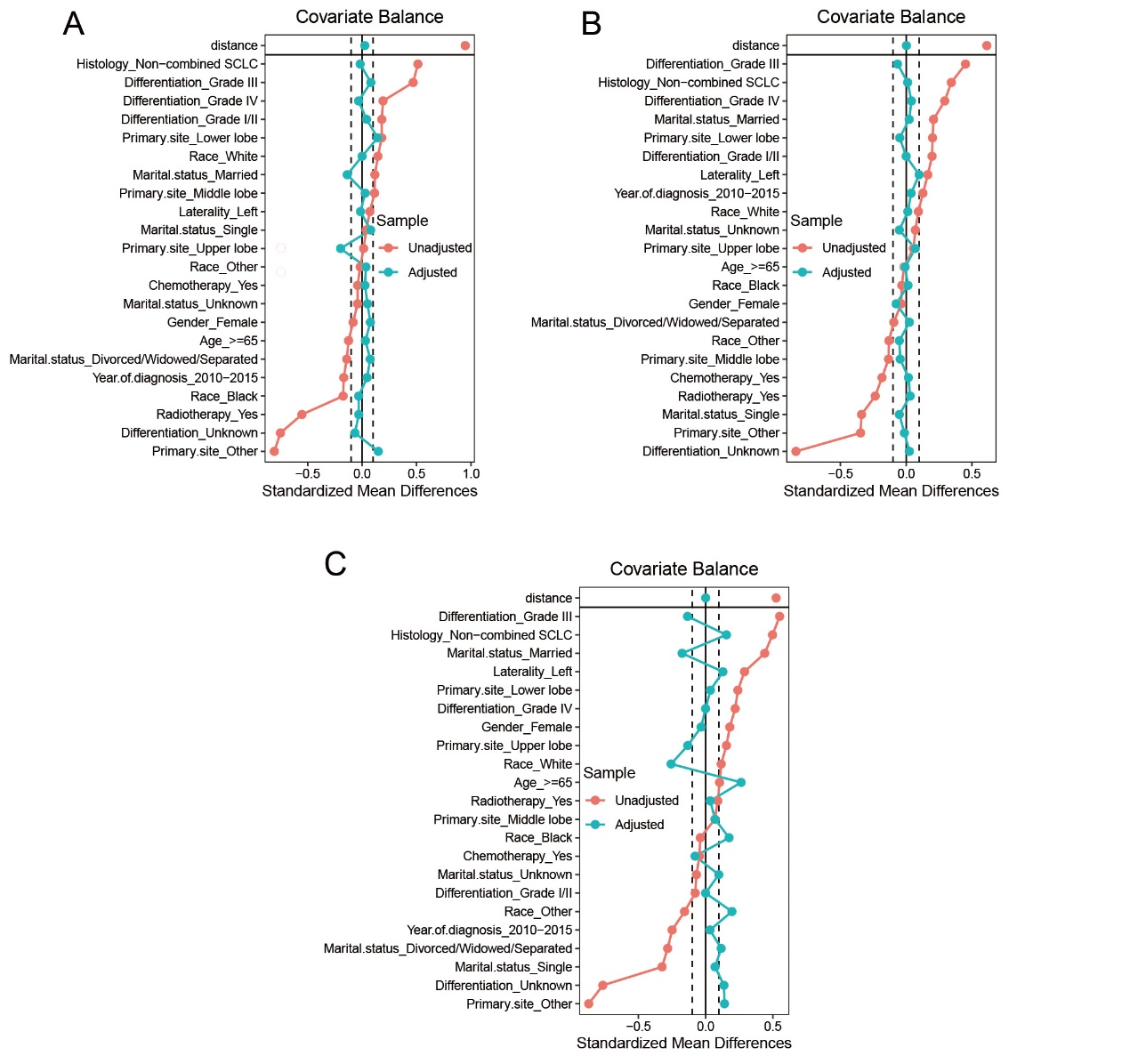


**Supplementary Fig. 2** SMD between surgery and no surgery in patients with stage IIB (A), stage IIIA (B), and stage IIIB (C) before and after PSM. The dotted lines denote the SMD of -0.1 and 0.1. SMD, standardized mean difference; PSM, propensity score matching.


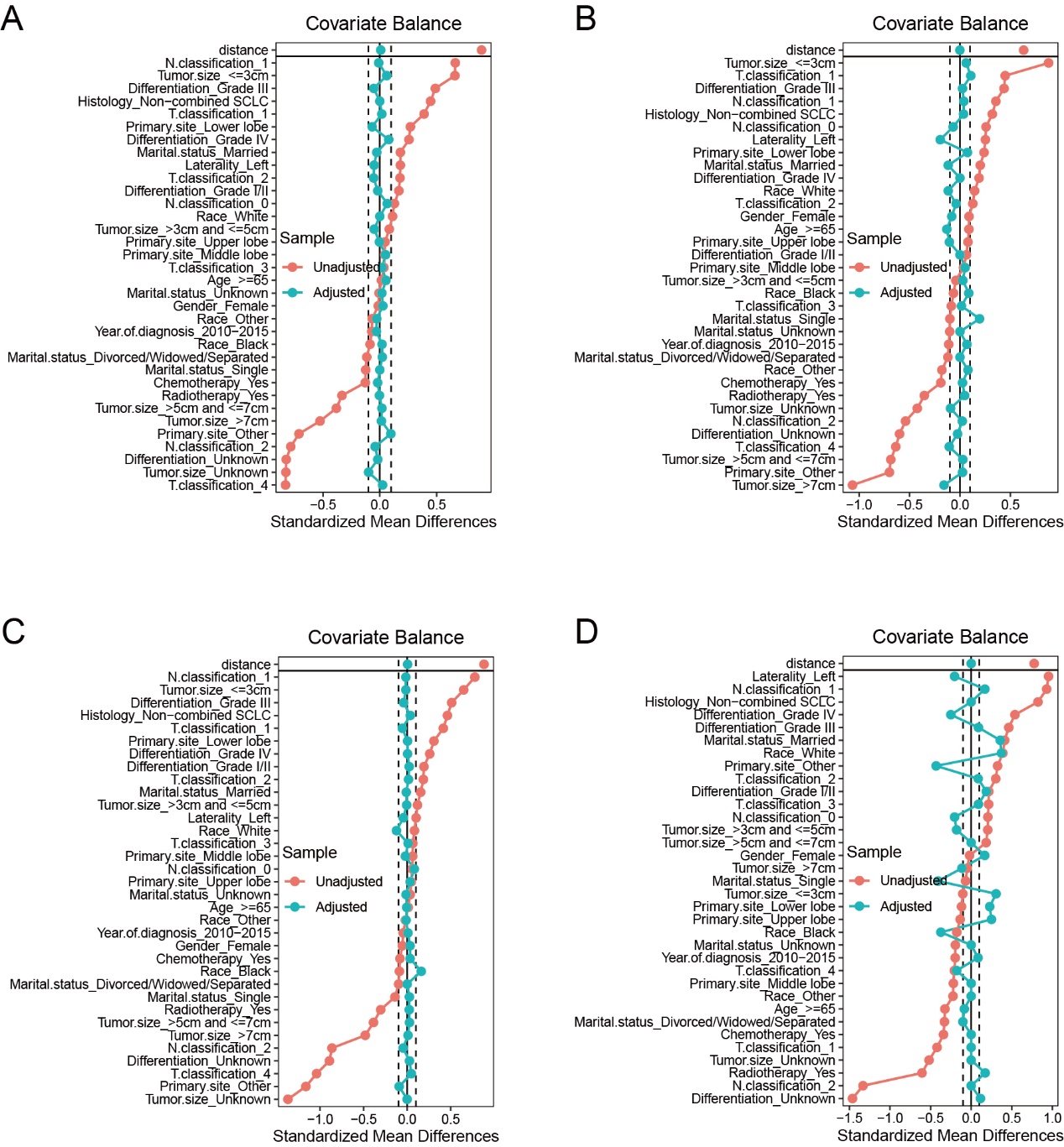


**Supplementary Fig. 3** SMD between surgery vs. no surgery (A), sublobectomy vs. no surgery (B), lobectomy vs. no surgery (C), and pneumonectomy vs. no surgery (D) in patients with stage T3-4N0M0/T1-4N1-2M0 SCLC before and after PSM adjusting tumor size, T classification, N classification and other baseline characteristics. The dotted lines denote the SMD of -0.1 and 0.1. SMD, standardized mean difference; PSM, propensity score matching.


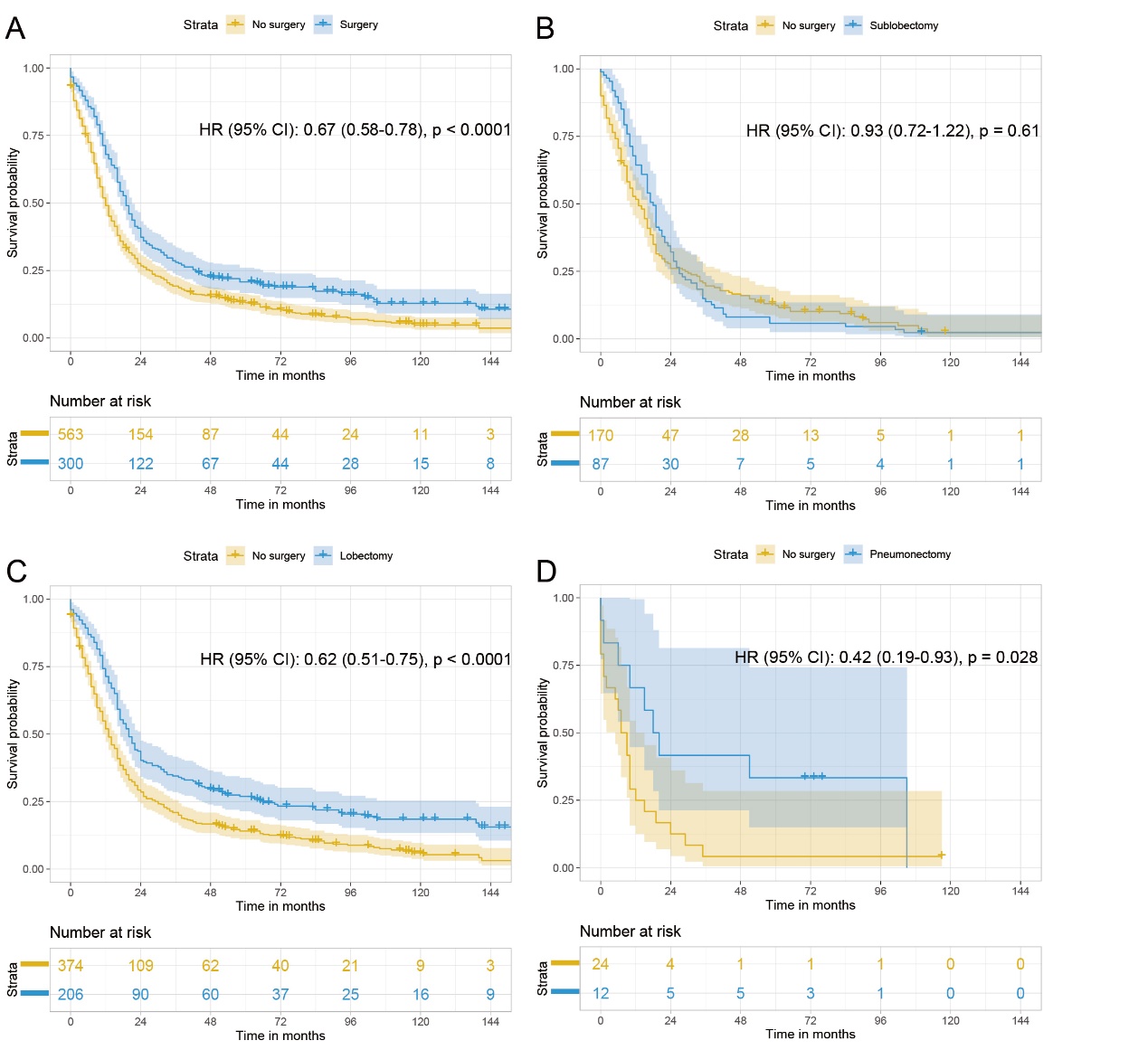


**Supplementary Fig. 4** Kaplan-Meier curves of OS for stage T3-4N0M0/T1-4N1-2M0 SCLC comparing surgery vs. no surgery (A), sublobectomy vs. no surgery (B), lobectomy vs. no surgery (C), and pneumonectomy vs. no surgery (D) after PSM adjusting tumor size, T classification, N classification and other baseline characteristics. OS, overall survival; SCLC, small cell lung cancer, PSM, propensity score matching.


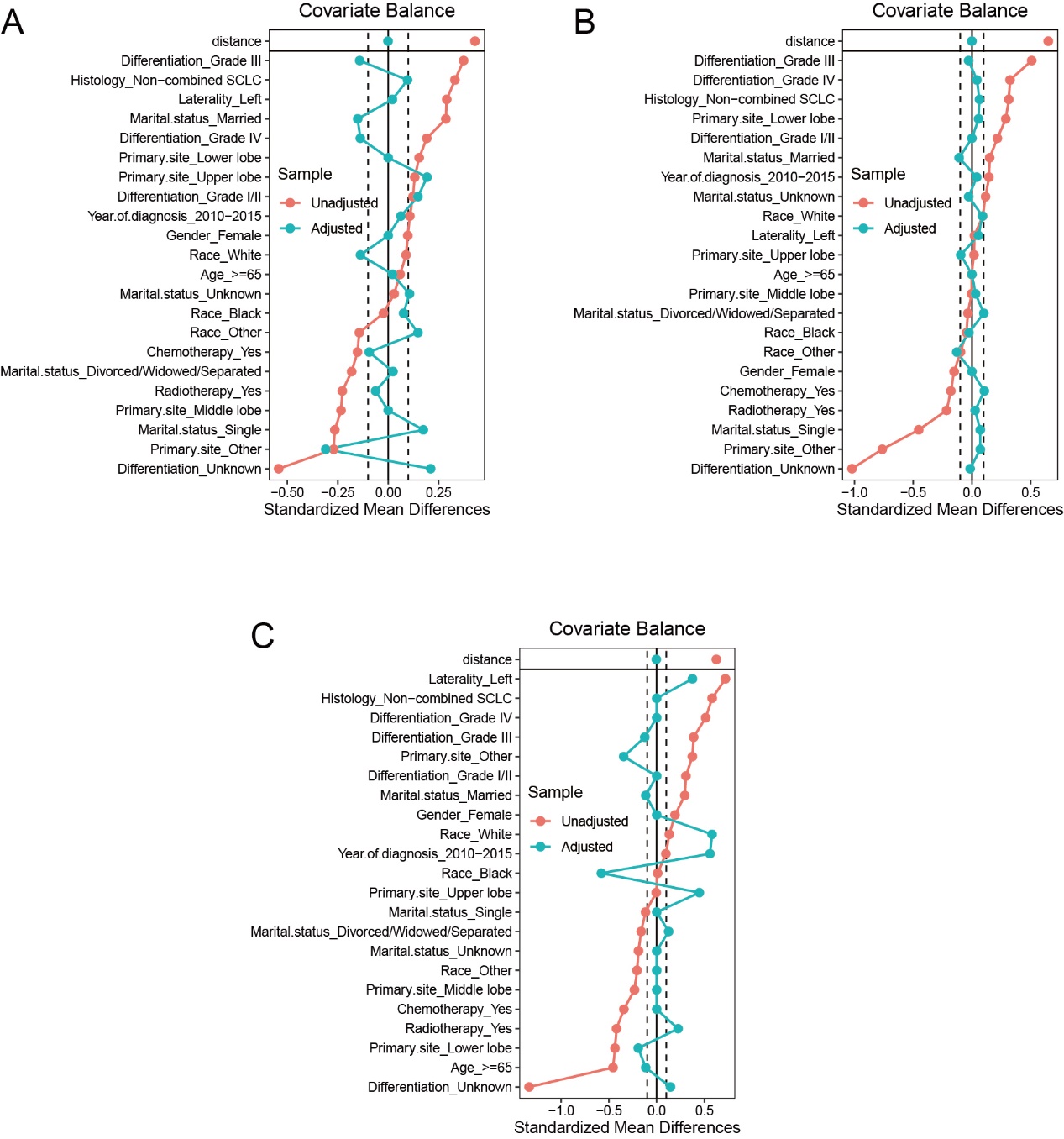


**Supplementary Fig. 5** SMD between sublobectomy vs. no surgery (A), lobectomy vs. no surgery (B), and pneumonectomy vs. no surgery (C) in patients with stage IIIA SCLC before and after PSM. The dotted lines denote the SMD of -0.1 and 0.1. SMD, standardized mean difference; PSM, propensity score matching.


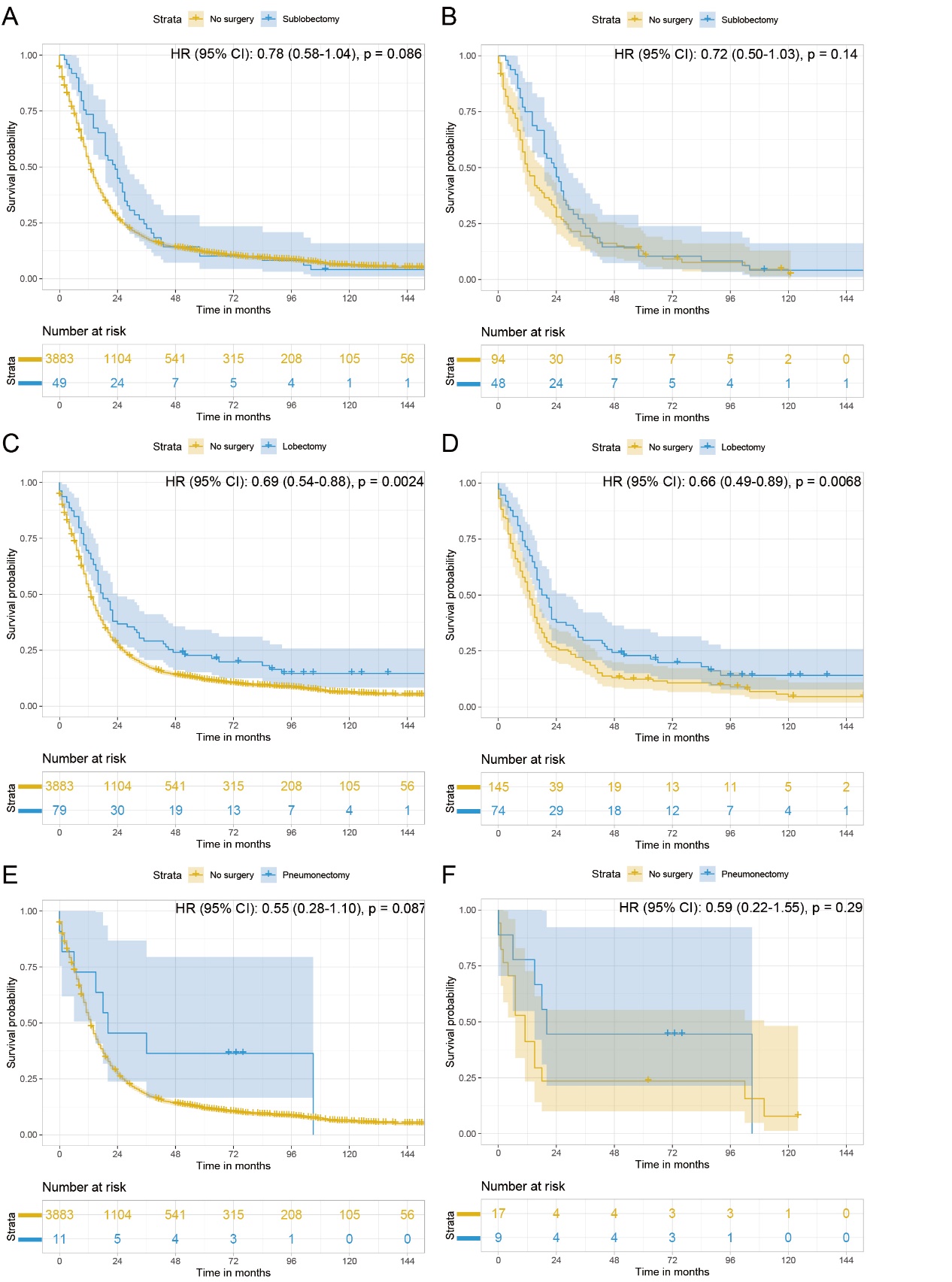


**Supplementary Fig. 6** Kaplan-Meier curves of OS for stage IIIA SCLC comparing sublobectomy vs. no surgery (A, B), lobectomy vs. no surgery (C, D), and pneumonectomy vs. no surgery (E, F) before and after PSM. OS, overall survival; SCLC, small cell lung cancer, PSM, propensity score matching.


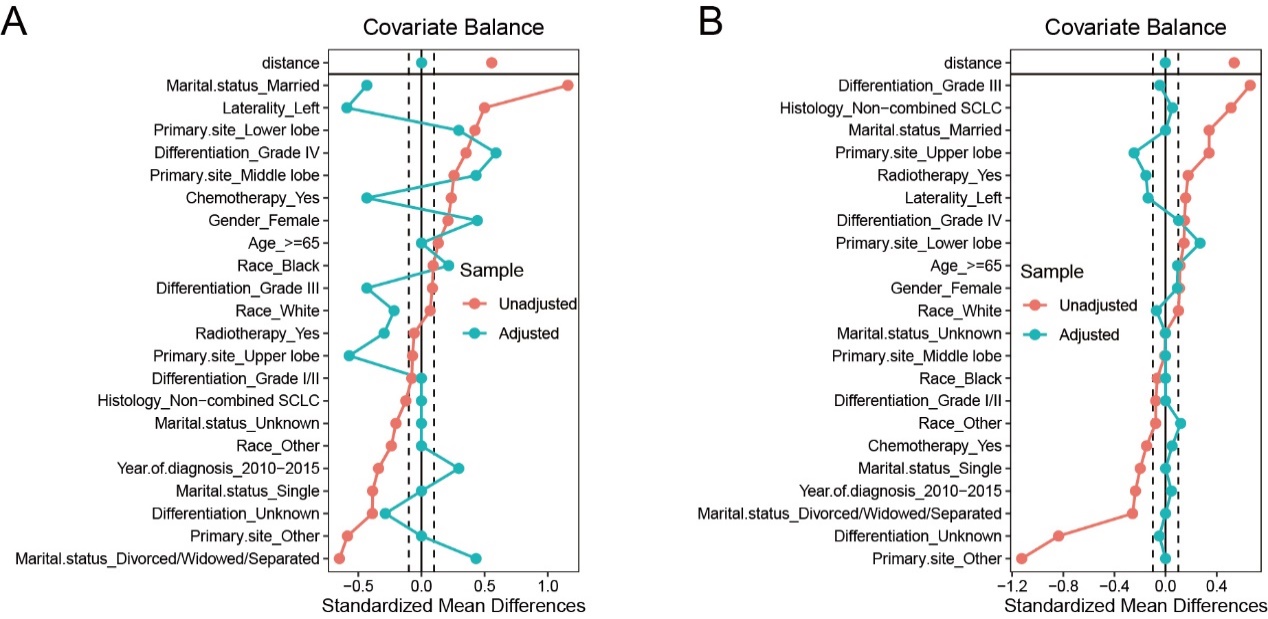


**Supplementary Fig. 7** SMD between sublobectomy vs. no surgery (A) and lobectomy vs. no surgery (B) in patients with stage IIIB SCLC before and after PSM. The dotted lines denote the SMD of -0.1 and 0.1. SMD, standardized mean difference; PSM, propensity score matching.


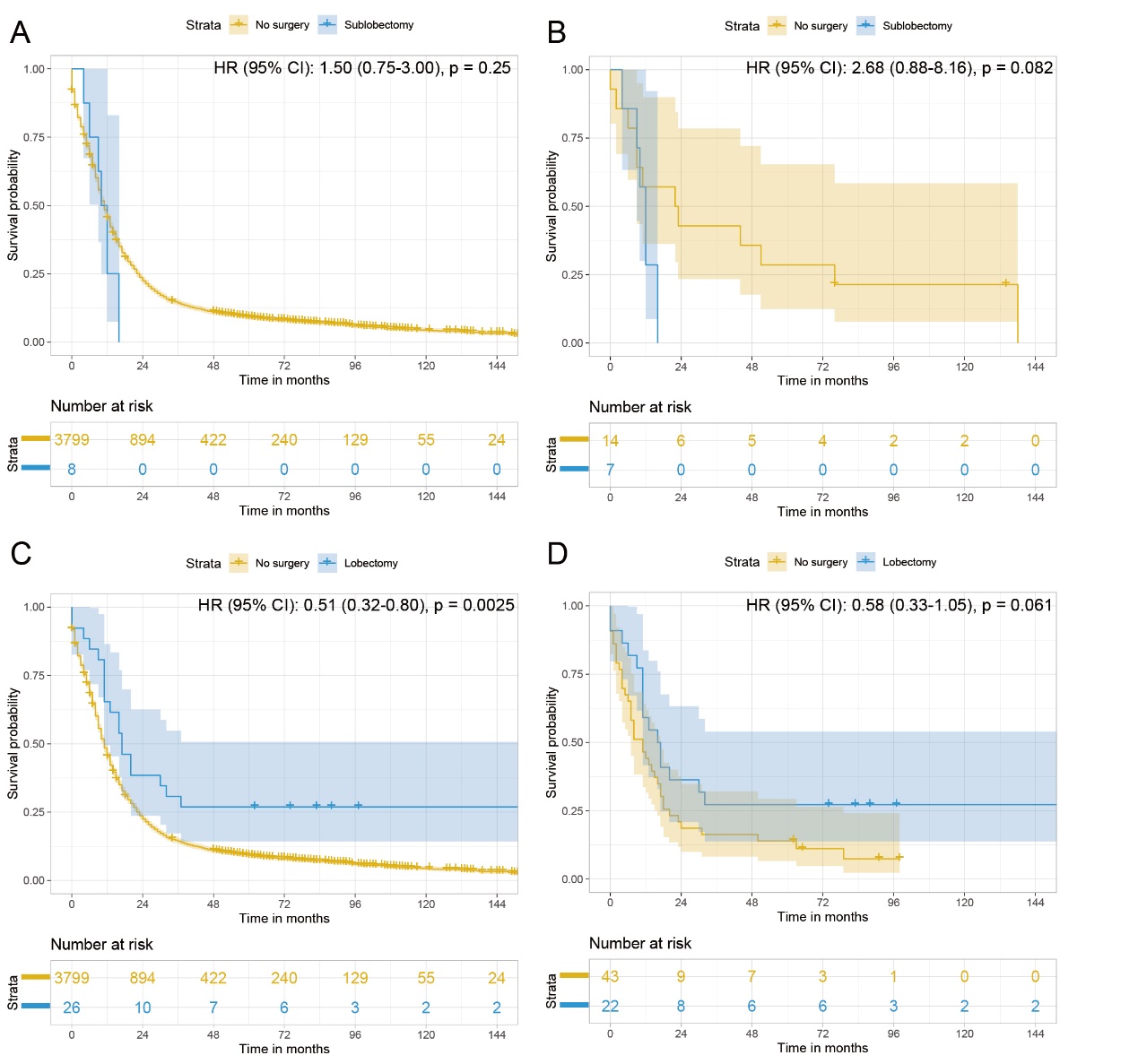


**Supplementary Fig. 8** Kaplan-Meier curves of OS for stage IIIB SCLC comparing sublobectomy vs. no surgery (A, B) and lobectomy vs. no surgery (C, D) before and after PSM. OS, overall survival; SCLC, small cell lung cancer, PSM, propensity score matching.
